# Notable Aspects of Glycan-Protein Interactions

**DOI:** 10.3390/biom5032056

**Published:** 2015-09-01

**Authors:** Miriam Cohen

**Affiliations:** Depatment of Cellular and Molecular Medicine, University of California, San Diego, 9500 Gilman Drive, BRF2 MC 0687, La Jolla, CA 92093-0687, USA; E-Mail: micohen@ucsd.edu; Tel.: +1-858-534-1346

**Keywords:** sperm, leukocytes, influenza A, FimH, mucus, cumulus oophorus, hyaluronan, rolling adhesion, stick and roll, surfacing, glycans, sialic acids

## Abstract

This mini review highlights several interesting aspects of glycan-mediated interactions that are common between cells, bacteria, and viruses. Glycans are ubiquitously found on all living cells, and in the extracellular milieu of multicellular organisms. They are known to mediate initial binding and recognition events of both immune cells and pathogens with their target cells or tissues. The host target tissues are hidden under a layer of secreted glycosylated decoy targets. In addition, pathogens can utilize and display host glycans to prevent identification as foreign by the host’s immune system (molecular mimicry). Both the host and pathogens continually evolve. The host evolves to prevent infection and the pathogens evolve to evade host defenses. Many pathogens express both glycan-binding proteins and glycosidases. Interestingly, these proteins are often located at the tip of elongated protrusions in bacteria, or in the leading edge of the cell. Glycan-protein interactions have low affinity and, as a result, multivalent interactions are often required to achieve biologically relevant binding. These enable dynamic forms of adhesion mechanisms, reviewed here, and include rolling (cells), stick and roll (bacteria) or surfacing (viruses).

## 1. Introduction

**Figure 1:**
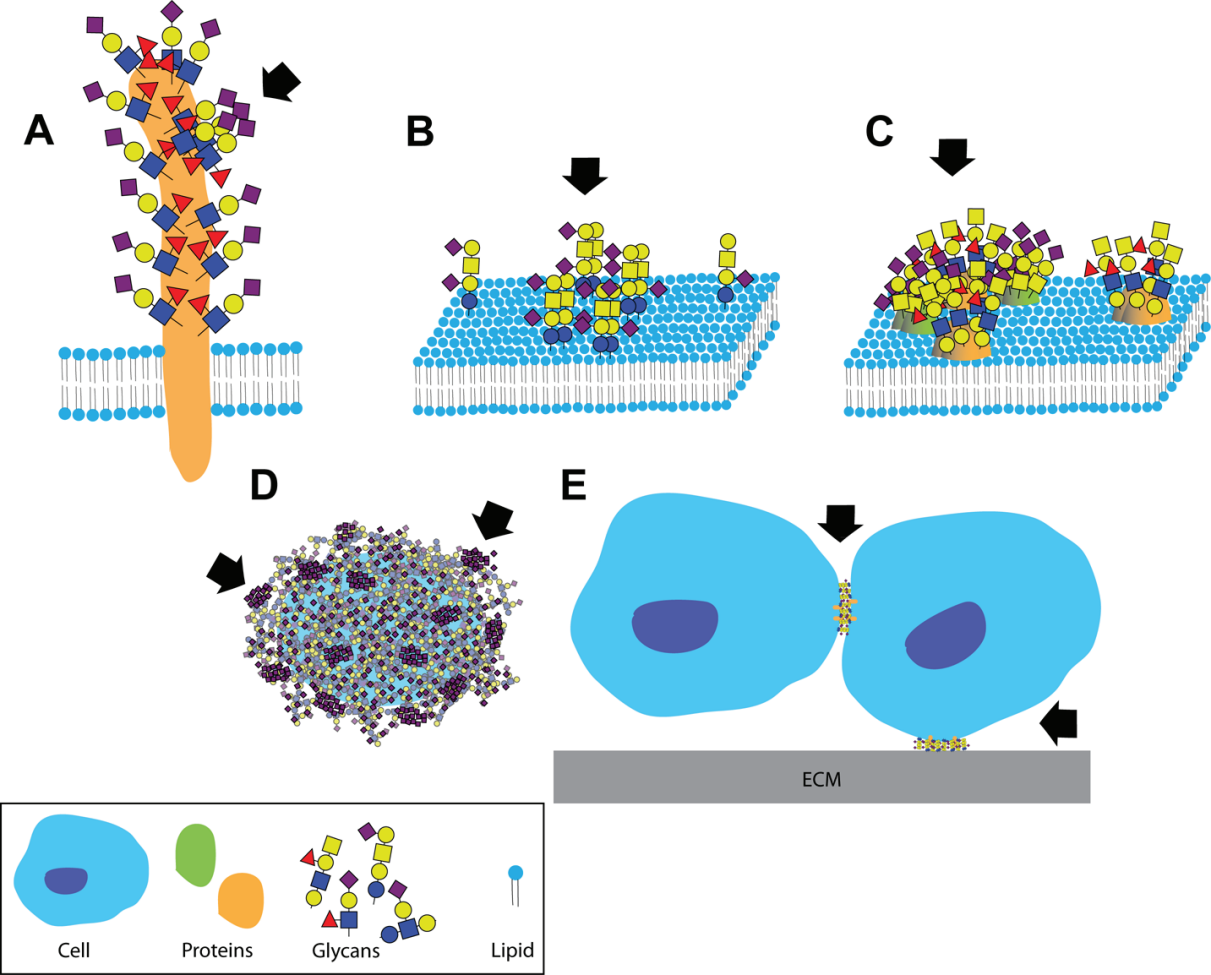
Clustered saccharide patches exist at spatial scales spanning several orders of magnitude. Clustered saccharide patches may form on (**A**) heavily glycosylated proteins; (**B**) on cell membranes due to interactions between two or more glycosphingolipids; (**C**) or glycoproteins; (**D**) Clustered saccharide patches can also form on pathogen polysaccharides; (**E**) Glycosynapses are clustered glycan microdomains, which mediate cell-cell and cell-extracellular matrix adhesion and signaling. Arrows indicate likely clustered saccharide patches. Figure reproduced with permission [6].

A dense and complex array of glycans covers the surface of all living cells in nature. In eukaryotic cells, glycolipids and glycoproteins are tethered to the plasma membrane; while glycosaminoglycans and mucins dominate the extracellular milieu (although glycoproteins are secreted to the extracellular matrix as well). This contributes to a highly glycosylated environment in which cellular interactions take place. It is, therefore, not surprising that glycans are involved in numerous aspects of cellular interactions with self and pathogens [1,2]. Glycans are the most diverse of the four fundamental building blocks of life (nucleic acids, amino acids, lipids, and glycans) [3]. Their biosynthesis and modifications are not template driven; rather they are the result of multiple enzymatic activities. Glycans can form linear or branched chains via either α or β glycosidic linkages to any available hydroxyl of another monosaccharide. These chains can be free (e.g., hyaluronan and milk oligosaccharides), attached to proteins (glycoproteins and proteoglycans) or attached to lipids (glycolipids). In addition, a range of modifications can be found on each of the glycosylation sites, leading to a phenomenon known as microheterogeneity [4,5]. Furthermore, the enormous diversity of unique glycan structures can be spatially organized into functionally meaningful clustered saccharide patches. There is ample evidence for the formation of clustered saccharide patches on heavily glycosylated proteins, at the plasma membrane or on capsular polysaccharides of pathogens. These clusters form specific ligands for specific glycan-binding proteins (Figure 1) [6].

Initial recognition and binding at the cell surface is often mediated by glycans not only due to their structural diversity, but also because of their quantity and dependence on avidity. Glycans are ideal molecules for mediating such interactions for several reasons. First, the high diversity of glycan modifications in conjunction with their spatial organization in clustered saccharide patches generates a unique “topology” for each protein, cell and tissue [4,6,7]. This provides high specificity for glycan-protein interactions. Despite their high abundance in tissues, glycan-mediated interactions occur only when the correct conformation or cluster is present. Cells with similar glycan content can display unique clustered saccharide patches and, as a result, can be differentially recognized by glycan-binding proteins [8]. The plasma membrane is both fluidic and dynamic; redistribution of glycoconjugates suffices to disperse or to form functional glycan clusters. Similarly, clustering of glycan-binding proteins on the membrane can form docking sites for the glycans on a cell or a pathogen. Perhaps the best example of this is the formation of 100–200 nm microdomains of *C*-lectins, which are the binding sites for viral glycans [9]. Second, high abundance of glycans on cells and in tissues increases the probability that interactions will occur. When a cell or a pathogen approaches the target tissue, it is often found in a fluid media (e.g., blood, mucus or urine) and under shear force [10]. Glycan binding proteins must be at close proximity to their ligands to achieve binding. The likelihood of a protein to encounter, and bind to its glycan counterpart is increased by having multiple repeating glycan structures on the target tissue. Third, the affinity of a single glycan-protein interaction is typically low (mM–μM *K_d_* values), rendering the interactions readily reversible. This allows quick release from the wrong target with a small energy penalty. Fourth, multivalent binding is often required in order to generate biologically relevant interactions. This directly results from the low binding affinity of a single glycan to the glycan-binding pocket of the protein. The requirement for multivalent binding contributes to the reversibility and to the specificity of glycan interactions. Association and dissociation of multivalent interactions, results in lateral movement on tissues prior to formation of firm adhesions. This is a phenomenon that is best described for hematopoietic cells [10].

**Figure 2:**
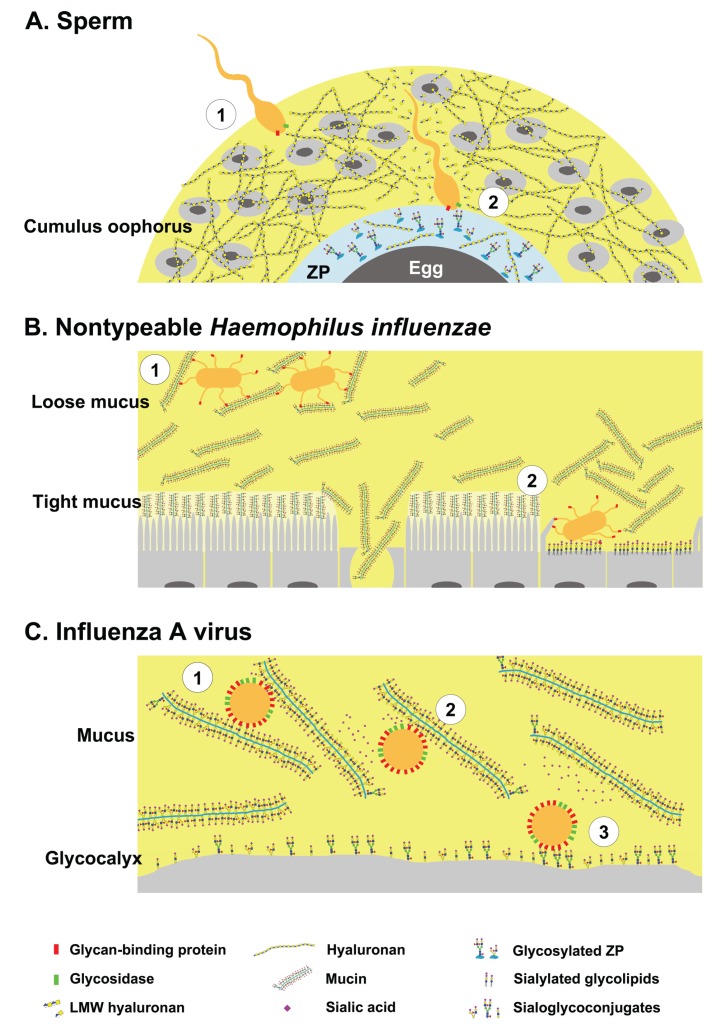
Glycans have dual roles as ligands and decoys during pathogen invasion. (**A-1**) Sperm bearing hyaluronan binding proteins and hyaluronidase negotiates the cumulus oophorus; (**A-2**) By cleaving hyaluronan, the sperm penetrates the cumulus layer, then binds to specific glycans on *zona pellucida* proteins; (**B-1**) Nontypeable *Haemophilus influenzae* (NTHi) binds glycosylated mucus and remains in the loose mucus layer; (**B-2**) Impaired mucus clearance due to ciliary or tissue damage enables NTHi binding to sialylated glycolipids on host cells; (**C-1**) Influenza A virus bearing hemagglutinin (HA) and neuraminidase (NA) binds to host mucins; (**C-2**) The virus can free itself from mucins by cleaving sialic acids; (**C-3**) and binds to sialoglycoconjugates on the host cells. Yellow shaded area represents the highly hydrated cumulus and mucus layers.

**Figure 3:**
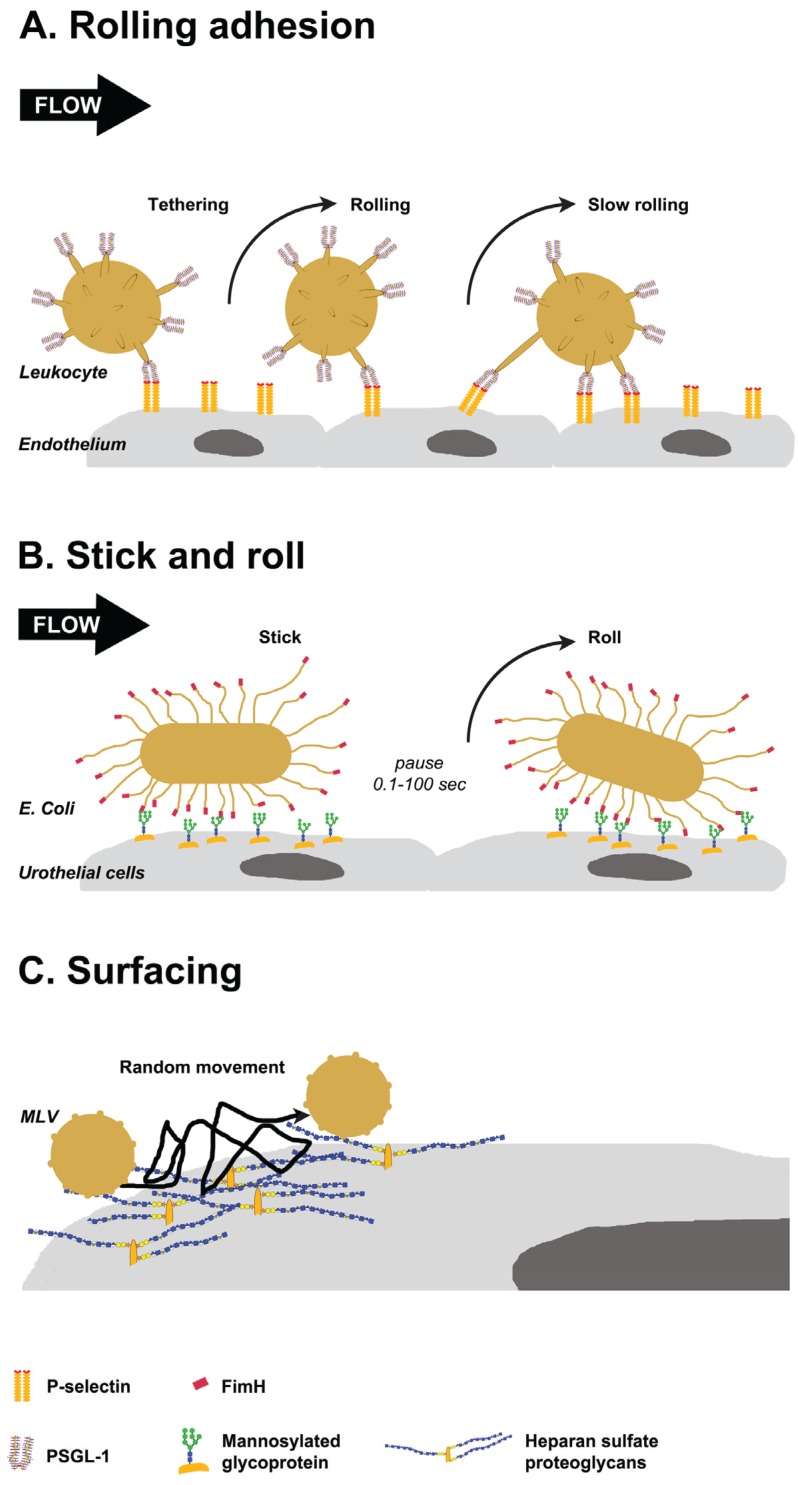
Glycan-mediated lateral movement on host tissues. (**A**) PSGL-1, localized at the tip of leukocyte villi, binds to P-selectin on the endothelium (tethering). Leukocytes pause when bonds form and move in the direction of flow when they break. New bonds are formed in the leading edge, resulting in rolling and slow rolling on the endothelium; (**B**) FimH proteins localized to the tip of *E. coli* pili interact with oligomannose on urothelial cells. The interactions persist for 0.1 to 100 s, then bacteria detaches and rolls on the cells. Switching between detaching and rolling leads to stick and roll adhesion; (**C**) Weak interactions of murine leukemia virus (MLV) with glycosaminoglycans on the cell surface mediate multidirectional movement with frequent jumps on the cell surface. This surfacing movement does not depend on the cytoskeleton.

This mini-review brings together a few notable concepts of glycan-mediated interactions, and is not meant to be an exhaustive survey of the literature. Clustered saccharide patches, and the many roles of glycans in host defense via pathogens-associated and self-associated molecular patterns, have been reviewed elsewhere [6,11,12]. This mini-review will focus on how the complex nature of glycan interactions forces pathogens or invading cells to develop strategies to overcome decoys and identify targets (Figure 2). Glycans are targeted as host receptors by invading pathogens, but are also presented as defensive decoys by the host to prevent infection. Another interesting aspect that will be discussed is how multivalent interactions under shear stress conditions can lead to lateral movement on the host tissue or cells (Figure 3). Adhesive interactions *in vivo* often occur under flow, and involve the capturing of a moving particle. Heavily glycosylated glycoconjugates typically extend from the plasma membrane and are suitable to mediate these interactions. Unfortunately, the very same characteristics that make glycans ideal molecules for initiating cellular interactions also pose technical challenges for investigating glycan interactions. Glycans form meaningful three-dimensional structures on proteins and on the plasma membrane, which have a biological function. However, three dimensional crystal structures and technologies for high-resolution imaging of glycans are not yet available. In essence, glycobiology is currently facing similar challenges that proteomics faced in the pre-crystallization era. Glycan structure is often studied by fragmenting glycoconjugates for analysis in mass spectrometry, and glycan binding is often tested on purified oligosaccharides bound to a solid surface.

## 2. Dual Role of Glycans during Pathogen Invasion—Receptors and Decoys

The cell surface receptors for many pathogens, from viruses to protozoa, are glycoconjugates [13,14]. In order to interact with host glycoconjugates, the pathogens need to be in close proximity to the plasma membrane. For pathogens that target the gastrointestinal, respiratory or female reproductive tracts, successful infection requires penetrating through glycosylated mucus secretions [15,16,17]. In an analogous manner, sperm cells need to successfully penetrate the glycosylated cumulus mass surrounding the oocyte in order to bind to the *Zona pellucida* glycoproteins and fertilize the egg [18]. Thus, host glycans have a dual role of being ligands on the plasma membrane and decoys in host secretions. Presented here are examples of a cell (sperm), bacteria (nontypeable *Haemophilus influenzae*) and virus (influenza A virus) that encounter glycosylated barrier that masks the glycan receptors of host cells.

### 2.1. Sperm-Egg Interactions

Fertilization is essential for sexually reproducing organisms. In mammals the highly glycosylated sperm travel in the female reproductive tract, where they undergo significant modifications, many of which are glycan-mediated [19]. Sperm are transferred from the male reproductive tract to the female vagina and then move through the cervix into the uterus. In most mammalian species, sperm are temporarily arrested in the lower oviduct before fertilization. Many mechanisms exist to prevent polyspermy including gradual release of the sperm from the oviductal sperm reservoir. As a result, the sperm-to-egg ratio in the ampulla is very low, and possibly only a handful of sperm reach the egg [20,21]. The ovulated egg is coated with a glycocalyx comprised of an interwoven mesh of *zona pellucida* (ZP) glycoproteins and the glycosaminoglycan hyaluronan [22]. In addition, the ZP layer is surrounding the cumulus oophorus, which is a substantial layer of cumulus cells embedded in copious amounts of hyaluronan [23]. The cumulus oophorus participates in stimulating sperm motility, and secretes lectins and chemoattractants that guide the sperm toward the egg [24,25].

For fertilization to occur a sperm cell needs to reach the egg, adhere, and fuse with the plasma membrane. To do so, the sperm must penetrate the hyaluronan-rich cumulus mass and bind to glycans that are presented on the ZP proteins. Then, the sperm must transit through the ZP layer into the perivitelline space where it fuses with the oocyte membrane via multivalent protein-protein interactions [26]. Interestingly, the sperm membrane contains both hyaluronan-binding proteins (HABP1, RHAMM) [27,28,29] and GPI-anchored hyaluronidase (Ph-20) [30,31]. In mice, sperm release Hyal5, another GPI anchored hyaluronidase, upon acrosome reaction [32]. The sperm binds to hyaluronan in the cumulus mass, probably via interactions of the hyaluronan-binding proteins. It has been suggested that hyaluronan binding improves motility and induces calcium release by sperm [33]. Because hyaluronan binding is important for fertilization, it is common practice to assess the ability of sperm to bind to hyaluronan in the *in vitro* fertilization (IVF) clinics. However, the sperm hyaluronidase activity is required for penetrating the cumulus layer [31]. The hyaluronidase Ph-20 is the major contributor to the digestion of cumulus hyaluronan [34,35], and it is at least 10-fold more active compared with testicular hyaluronidase [31]. It is conceivable that such binding-cleaving interaction with cumulus hyaluronan contributes to the translocation of sperm through the cumulus layer. Once the sperm penetrates the cumulus layer, it binds to specific glycans and/or peptide sequence on the ZP glycoproteins [36]. In humans, the interactions are primarily mediated by binding to sialyl-Lewis X (Neu5Acα2-3Galβ1-4(Fucα1-3)GlcNAcβ1-R, SLe^x^) on the ZP proteins, in a sialic acids dependent manner [37]. Although several putative proteins have been suggested, it is not clear which sperm proteins mediate these interactions [38].

### 2.2. Nontypeable Haemophilus Influenzae and Host Mucins

Bacteria have complex interactions with the host mucus layer. Mucosal cells are coated with a tight impenetrable layer of tethered mucus, covered with a second layer of loosely adherent mucus that is highly dynamic. The mucus is composed of secreted mucin glycoproteins, nonspecific antimicrobials (e.g., defensins, cystatins), and specific antimicrobial immunoglobulins [39,40]. Most bacteria express more than one type of glycan-binding proteins (adhesins) and glycosidases [41]. Some bacteria can bind mucins to colonize the mucus while the glycosidase activity allows the bacteria to obtain nutrients by foraging hexose sugars from the host diet or cleaving mucin glycoproteins [41,42]. However, mucus forms an important protective barrier between pathogens and the underlying host tissue [16,43]. Opportunistic commensal bacteria such as *Haemophilus influenzae* can infect areas of damaged epithelium due to impaired mucus clearance [44].

Nontypeable (non-encapsulated) *H. influenzae* (NTHi) are human specific gram-negative bacteria that colonize the upper airways, and are the common cause for respiratory tract and ear infections [45]. Several adhesins, including hemagglutinating pili, P2 and P5, on the NTHi outer membrane, bind to sialylated *O*-linked glycans on mucins [46,47]. NTHi binding to secreted mucins inhibits infection of underlying cells [46]. NTHi scavenges free sialic acids from the host that can be utilized as a nutrient source or incorporated into lipooligosaccharides on the bacterial membrane [48]. Sialic acids incorporated into the bacteria lipooligosaccharides allow NTHi to mimic the molecular signature of the host and aids in manipulation of the host immune system [49,50]. To facilitate infection the HMW1 protein of NTHi binds to gangliosides and sialylated lacto/neolacto glycolipids on the host cells [51,52]. In addition, NTHi can bind to sulfated glycosaminoglycans of the host [53]. Interestingly, despite binding to and utilizing host sialic acids, NTHi does not express sialidase [54]. How does NTHi get released from the sialylated mucins and infect underlying cells? NTHi infection typically occurs when mucus clearance is impaired due to damaged epithelium and lack of ciliary movement [45]. Cellular damage can occur due to events unrelated to the bacteria, however, NTHi secretes factors that cause slowing and detachment of cilia [45,55]. Mucus clearance is imperative for the protective efficacy of the mucus layer [15], thus impaired mucus clearance may enable NTHi to penetrate the mucus layer and infect underlying cells.

### 2.3. Influenza A Viruses and Host Mucins

There are numerous examples of viruses that initiate infection by binding host glycans. *Herpes simplex* virus binds the glycosaminoglycan heparan sulfate [56]. Most genotypes of the *Norovirus* bind to the oligosaccharides defining the histo-blood group antigens (ABH and Lewis) [57]. Perhaps the best example of the dual role of glycans in host-pathogen interactions is *influenza A* virus binding to sialylated host glycans [58]. Respiratory pathogens, such as the influenza viruses, encounter a thick layer of mucus that masks the target epithelial cells. Influenza A virus is an enveloped RNA virus belonging to the family of *Orthomyxoviridae.* The virus envelope has two major glycoproteins: Sialic acid-binding hemagglutinin (HA) and sialic acid-cleaving neuraminidase (NA). A single virion is estimated to have 500–1000 HA trimers and 100–500 NA tetramers on the envelope [59,60]. For an average virion with 100 μm diameter, the density of 500 HA molecule corresponds to 15,000 HA trimers per μm^2^. However, HA and NA are not evenly distributed in the membrane but are clustered [61], creating high local density of these proteins.

When the virus encounters the host mucus layer, sialylated mucins can act as decoy ligands for the influenza HA and provide the first line of defense from influenza infection [16,62]. Mucins contain a variable number (21–125) of 20 amino acids tandem repeats, each with five potential sites for *O*-glycosylation on serine or threonine residues. Depending on the mucin and the cell type, between two to five of the glycosylation sites can be occupied simultaneously [63]. This translates into a large cluster of 42 to 625 *O*-glycans, which contribute to 70% to 80% of the mucin weight. Such high density of sialic acids increases the probability of multivalent interactions between virus HA and the mucins. Influenza A neuraminidase activity releases the virus from the mucins [62,64]. Along with the neuraminidase activity of NA, the low affinity of interactions between the virus HA and the host sialic acids aids in dissociation of the virus from the decoy target. The HA proteins were shown to have very high affinity for *N*-linked sialoglycans [65]. Furthermore, it was also recently demonstrated that influenza A binds to *N*-linked glycans on microarray at very high affinity [66,67]. *N*-linked glycans are only a small fraction of mucins glycans, but are highly abundant on the plasma membrane. It is possible that influenza HA affinity to sialoglycoconjugates on the plasma membrane is higher compared with the affinity to sialylated mucins. This could be an important adaptation of the virus to evade decoy glycans of the host.

## 3. Glycan-Mediated Lateral Movement on Host Tissues

Non-simultaneous association and dissociation of low affinity single protein-glycan interaction may result in a lateral movement of virus, bacteria or cell upon initial engagement with the host tissue. These initial dynamic interactions will be followed by the formation of strong protein-protein interactions, signaling or internalization. Adhesive interactions with host tissue often occur under flow-induced shear generated by ciliary beating, urinary flow, tears or blood. Flow-induced shear can prevent attachment and wash off bound pathogens or cells especially when the adhesive interactions are weakened under shear force (slip bonds). In contrast, certain protein-glycan interactions become stronger under shear force, forming “catch bonds” which mediate tethering and rolling adhesion [68]. This type of adhesion is well characterized for hematopoietic cells, white blood cells, and platelets (see the excellent review by McEver and Zhu, 2010 [10]). A similar form of adhesion, termed stick-and-roll, is mediated by the bacterial FimH protein [68]. Host glycosaminoglycans mediate lateral movement of viruses on host cells in a process termed “surfacing” [69].

### 3.1. Selectin-Mediated Leukocyte Rolling

Circulating leukocytes adhere to a damaged or activated endothelium in the blood vessels wall. Rapid adhesion kinetics is necessary to initiate contact between flowing cells and cells on the vessel wall. Tethering can occur when adhesion receptors contact their ligands. This process is mediated by selectins and their glycoprotein ligands. Inflamed endothelial cells express P-selectin and E-selectin, which are Ca^2+^ dependent lectins (*C*-type lectins). Leukocytes present clusters of the P-selectin glycoprotein ligand-1 (PSGL-1) in lipid rafts at the tips of their microvilli [70,71]. In addition, the E-selectin ligand-1 (ESL-1) [72], and CD44 are presented along the microvilli and at the valleys between microvilli, respectively [73]. The latter glycoproteins are ligands for E-selectin [10,74]. The predominant ligand for P-selectin is the dimeric mucin type glycoprotein PSGL-1. PSGL-1 has a densely *O*-glycosylated extracellular domain and contains up to three sulfated tyrosine residues at the terminal end [75,76,77,78,79]. L-selectin is localized to the tips of leukocyte microvilli and binds to sulfated *O*-glycans on peripheral node addressin (PNAD), or to PSGL-1 on other leukocytes [10,80]. It is interesting to note that P- and L-selectin bind to a clustered saccharide patch comprised of a sulfates and *O*-linked sialyl Lewis X in a spatial display that is unique for each of them [6,81].

Leukocytes circulating in the blood vessel are subjected to laminar flow. The first step of leukocytes interactions with the endothelium is a capture (tether) mechanism. Selectins need to be in close proximity of their ligand to form an interaction. Fast moving cells make frequent collisions with the vessel wall, increasing the chance to interact with selectins on the endothelial cells [10]. The localization of PSGL-1 and L-selectins at the microvilli tip, which is extended 0.2–0.3 µm from the cell surface, greatly enhances their ability to form tethers under shear force [73,82]. Furthermore, when the interactions last long enough, an individual microvilli can stretch into very long tethers [82]. Both selectins and PSGL-1 are extended molecules, with binding domains at the distal *N*-terminus. This architecture enhances tethering and rolling by increasing the probability of protein interactions. Both PSGL-1 and P-selectin form non-covalent dimers and are clustered in membrane domains, which increases multivalent interactions and reduces the force on individual bonds [10].

Once captured, the leukocytes begin to roll on the endothelial cells. The rolling transduces signals from adhesion receptors and chemokine receptors that cause the cells to roll slower and eventually to stop. The low affinity nature of protein-glycan interactions is critical for rolling to occur, and also explains why shear force enhances the binding. Leukocytes pause when bonds are formed, and move in the direction of the flow when these bonds break. New bonds are formed at the leading edge to replace bonds that dissociate at the trailing edge [10]. Thus, the rapid on-off rate of selectins interactions with their ligands is required for leukocyte rolling. The measured *k_off_* for P-selectin interaction with PSGL-1, for example, is 1.4 s^−1^. However *k_on_* rate of (1 − 4) × 10^6^ M^−1^·s^−1^ results in high affinity at equilibrium (0.3–1.5 μM) [83].

### 3.2. FimH Mediated Stick-and-Roll Adhesion

Interactions of many bacteria with the host tissue occur in a dynamic fluid media and under flow-induced shear. Some bacteria can form catch bonds with the tissue, resulting in rolling adhesion behavior that is similar to selectin-mediated adhesion. Gram-negative bacteria mediate host binding by adhesins that are located at the tip of hair-like appendages called fimbriae (pili) [84]. Each fimbria is several micrometers long, and can stretch up to ten micrometer during adhesion [68]. The uropathogenic *E. coli* bacteria, which causes urinary tract infection in humans, has a single adhesin, termed FimH, at the tip of each fimbria [85]. FimH is an elongated molecule comprised of two subunits: A fimbria attached pilin domain and a lectin domain that binds to terminal mannose residues [68]. Terminally exposed mannose residues (Man_6_GlcNAc_2_ to Man_9_GlcNAc_2_) on urothelial cells are found in high levels on uroplakin Ia and the Tamm-Horsfall glycoprotein uromodulin [86,87,88].

Similar to selectin mediated rolling adhesion, FimH is located at the tip of an extended protrusion. In contrast to selectin mediated adhesion, multiple FimH proteins interact simultaneously with the host glycoconjugates. These multiple low-affinity interactions are enhanced under shear force due to conformational changes in the FimH protein [10]. In the absence of shear, the pilin and lectin domain of FimH tightly interact, resulting in low affinity to mannose [89,90]. Shear force leads to conformational changes that separate the pilin domain from the lectin domain, and the lectin domain adopts an elongated confirmation. In the open conformation, FimH affinity to mannose increases 200-fold. The dynamic structural change mediate catch bonds under shear force [91]. FimH interactions with mannosylated host glycans lasts substantially longer (seconds) compared with selectin interactions with their ligands (milliseconds). This allows for more than one FimH molecule to form catch bonds simultaneously. Thus, after tethering, the bacteria can immobilize for very short periods (100 ms) and very long periods (100 s). This results in switching between rolling and adhesion, leading to the term stick-and-roll adhesion. In addition, bacterial transition to firm adhesion does not require a second ligand (e.g., Integrin) and can revert from stationary back to rolling. The bacteria stops rolling at shear above 20 dynes/cm^2^. This may allow bacteria to preferentially bind in high shear microenvironments [68].

### 3.3. Heparan Sulfate Proteoglycans Mediated Surfacing

Heparan sulfate proteoglycans (HSPGs) are widely distributed within tissues, both in the extracellular matrix and on the cell membrane. The long negatively charged glycosaminoglycan polymers extend 20–150 nm from the plasma membrane [92]. Retroviruses, including Murine leukemia virus (MLV) and human immunodeficiency virus (HIV), are able to bind cells via interactions with HSPGs [93,94,95]. Similarly, Merkel cell polyomavirus can also infect the host by binding to HSPGs [96]. This is in contrast to other polyomoaviruses, including Murine polyoma virus (MPV) and simian virus 40 (SV40), which utilize gangliosides (sialylated glycolipids) as receptors to bind and infect the host cells [97,98]. Virus interactions with HSPGs have relatively weak affinity and can occur even in the absence of a specific viral receptor [69]. Interestingly, the weak interactions with HSPGs mediate rapid multidirectional movement with frequent “jumps” of the virus on the host plasma membrane, a phenomena termed “surfacing” [69]. Unlike virus “surfing”, which is actin dependent unidirectional lateral movement from the cell periphery to entry sites at the cell body [99,100,101,102], surfacing does not depend on the cytoskeleton of the host cell.

Surfacing adhesion was observed by single particle tracking of fluorescently labeled MLVs. Both exogenously added MLVs and by MLVs that were produced by chronically infected cells exhibit surfacing motility. This was inhibited by addition of heparin, or heparinase treatment of the host cell [69]. Similarly, such rapid lateral movement was observed when MPV-like particles were tracked on cells and isolated lipid layers. There is no evidence that MPV can bind HSPGs, or that heparin can inhibit the lateral movement. Depletion of cholesterol dramatically reduced the particles mobility, suggesting that it is mediated by interactions with gangliosides that are typically associated with lipid rafts [103]. Multivalent interactions are likely required to maintain virus surfacing on plasma membrane.

## 4. Concluding Remarks—Challenges in Investigating Glycan-Binding

The unique properties of glycan-mediated adhesion result in complex interactions of host cells with immune cells or pathogens. The low affinity of glycan-binding proteins to their respective glycosylated ligands leads to the formation of multivalent interactions in biological systems. This becomes even more complicated when the glycosylated ligand is in the form of a clustered saccharide patch. Investigation of these interactions requires creative approaches that consider multivalency, heterogeneity, and lateral mobility of natural glycans. One of the major advancements in glycan imaging technology was the introduction of metabolic labeling of glycan with bioorthogonal sugars (reviewed in [104,105]). This approach enabled *in vivo* imaging of glycans in live cells and living organisms. For the study of glycan recognition, glycan microarrays technology has proven to be very useful. In the past decade, this technology has rapidly evolved into diverse platforms that display a wide range of glycan structures that are both synthetic and biologically derived.

Several approaches have been successfully applied for increasing glycans multivalency on the microarrays. The glycans can be conjugated to a polymer core (glycopolymers) a protein core (neoglycoproteins), nanoparticles (glyconanoparticles), liposomes (glycoliposomes), or dendrimers (glycodendrimers) [106,107]. To address the issue of glycan heterogeneity in nature, glycans extracts from biological samples are purified, fluorescently tagged, and printed on the array. This shotgun glycan microarray may facilitate discovery of previously unknown glycans [108,109]. Lateral mobility of glycans in the plasma membrane can be addressed by fluidic arrays, in which glycans are presented on lipid bilayers [110], or on neoglycolipids that are printed on nitrocellulose-coated glass surfaces [111]. Development of current technologies and the emergence of new technologies will enable deeper understanding of the complex glycan interactions.
